# Pilot Study of F18-Florbetapir in the Early Evaluation of Cardiac Amyloidosis

**DOI:** 10.3389/fcvm.2021.693194

**Published:** 2021-06-25

**Authors:** Brett W. Sperry, Ashley Bock, Frank P. DiFilippo, Joseph P. Donnelly, Mazen Hanna, Wael A. Jaber

**Affiliations:** ^1^Cleveland Clinic Foundation, Kansas City, MO, United States; ^2^Saint Luke's Mid America Heart Institute, Kansas City, MO, United States; ^3^University of Missouri-Kansas City, Kansas City, MO, United States

**Keywords:** light chain amyloidosis, florbetaben, cardiomyopathy, technetium pyrophosphate, positron emision tomography, ATTR

## Abstract

**Background:** Cardiac amyloidosis is an increasingly recognized etiology of heart failure, in part due to the rise of non-invasive nuclear bone scintigraphy. Molecular imaging using positron emission tomography (PET) has promised the direct visualization of cardiac amyloid fibrils. We sought to assess the performance of F18-florbetapir PET in patients with a potential for cardiac amyloidosis in order to identify early disease.

**Methods:** We performed a pilot study of 12 patients: one with asymptomatic transthyretin cardiac amyloidosis, seven with a potential for developing cardiac amyloidosis (two smoldering myeloma and five with extracardiac biopsy demonstrating transthyretin amyloid deposits and negative technetium pyrophosphate scans), and four controls. Patients were imaged with PET/CT in listmode 10–20 min after receiving F18-florbetapir. Static images were created from this acquisition, and mean standardized uptake values (SUVs) of the left ventricular myocardium, blood pool, paraspinal muscles, and liver were calculated.

**Results:** All 12 patients demonstrated radiotracer uptake in the myocardium with mean SUV of 2.3 ± 0.4 and blood pool SUV of 0.8 ± 0.1. The patient with cardiac amyloidosis had SUV of 3.3, while mean SUV for patients at risk was 2.3 ± 0.4 and for controls was 2.2 ± 0.3. After 3 years of follow-up, one patient with SUV below the mean was subsequently diagnosed with ATTR cardiac amyloidosis.

**Conclusion:** In this cohort, PET with F18-florbetapir demonstrated non-specific radiotracer uptake in the myocardium in all patients using a static image protocol; though, the highest values were noted in a patient with ATTR cardiac amyloidosis. There was no difference in the intensity of F18-florbetapir uptake in at-risk patients and controls. Future studies should continue to investigate metabolic PET tracers and protocols in cardiac amyloidosis, including in early disease.

## Introduction

Cardiac amyloidosis is an increasingly recognized etiology of heart failure (1), in part due to the rise of non-invasive cardiac imaging such as echocardiography with longitudinal strain, nuclear bone scintigraphy, and cardiac magnetic resonance imaging. Echocardiography with longitudinal strain and cardiac magnetic resonance imaging may demonstrate findings consistent with cardiac amyloidosis and improve prognostication, while bone scintigraphy with technetium-based agent [pyrophosphate (PYP), 3,3-diphosphono-1,2-propanodicarboxylic acid (DPD), and hydroxymethylene diphosphonate (HMDP)] are highly specific for transthyretin cardiac amyloidosis in the setting of negative blood and urine testing for a plasma cell disorder (2). These modalities have typically been studied in symptomatic patients, though there is evidence that pre-symptomatic/early disease may be detected (3). Data for direct visualization of amyloid fibrils with amyloid-binding PET agents are emerging. Thioflavin analogs such as F18-florbetapir have shown good discrimination of quantitative uptake between symptomatic cardiac amyloidosis as compared to healthy controls (3). Additional studies have demonstrated non-cardiac uptake in patients with light chain (AL) amyloidosis, highlighting the systemic nature of that disease (4, 5). Though, differentiation between light chain (AL) and transthyretin (ATTR) cardiac uptake may not be possible (6).

F18-florbetapir is FDA approved and indicated for β amyloid imaging of the brain to estimate plaque density in adults with cognitive impairment who are being evaluated for Alzheimer's Disease. As this radiotracer has demonstrated high sensitivity in early disease, this pilot study sought to test F18-florbetapir for early detection of cardiac amyloid deposits in asymptomatic patients.

## Methods

A total of 12 patients were prospectively enrolled in this pilot study and followed for 3 years. One patient was considered to have ATTR cardiac amyloidosis, seven patients had a potential for development of cardiac amyloidosis (two smoldering myeloma and five with extracardiac biopsy demonstrating transthyretin amyloid deposits and negative technetium pyrophosphate scans), and there were four control patients. Control patients were selected as they had negative biopsies for amyloidosis during routine carpal tunnel release surgery without signs or symptoms of the disease (7). This study was approved by the Institutional Review Board and Ethics Committee at the Cleveland Clinic, and all patients signed informed consent.

Patients were imaged using F18-florbetapir (10mCi) using a Biograph mCT 128 (Siemens Healthineers, Malvern, PA) PET/CT scanner with lutetium oxyorthosilicate crystals, time-of-flight capable photomultiplier tubes with coincidence timing resolution of 550 ps, and PET detectors covering an axial field of view of 21.4 cm. Images were acquired in listmode from 10 to 20 min after radiotracer injection, and static images were reconstructed by summing data including 3D iterative time-of-flight with resolution modeling. This post-injection time was chosen based upon prior study showing a large differential in standardized uptake values (SUVs) between patients and controls (8). Images were interpreted by identifying myocardial radiotracer uptake qualitatively (present or absent) and quantitatively using Corridor4DM software (Invia, Ann Arbor, MI). For quantitative measures, PET images were reoriented along the standard cardiac axes and the mean and standard deviation of cardiac uptake in the entire myocardium was quantified using SUVs. Regions of interest were also drawn in the blood pool (left atrium), paraspinal muscles, and liver and mean SUVs were calculated.

PYP scintigraphy was performed using SPECT/CT with Siemens Symbia T6 cameras, and patients were imaged 3 h after infusion of 20 mCi+/– 10% of technetium PYP intravenously as previously described (9). A semiquantitative score and heart-to-contralateral lung ratios were obtained, and studies were considered positive if there was radiotracer uptake localized to the myocardium on SPECT/CT images (9).

## Results

**Figure 1:**
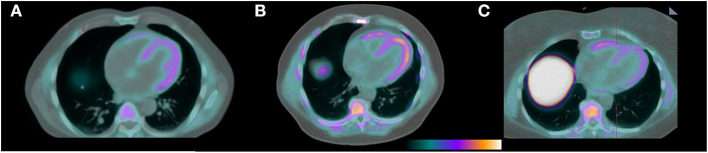
F18-florbetapir PET/CT images demonstrating identifiable myocardial uptake of radiotracer in the patient with confirmed cardiac amyloidosis by technetium PYP (**A**, mean myocardial SUV 3.3), a patient with light chain MGUS and a potential for having cardiac AL amyloidosis (**B**, mean myocardial SUV 2.7), and a control patient with a negative tenosynovial biopsy for amyloidosis at the time of carpal tunnel release surgery (**C**, mean myocardial SUV 2.0).

F18-florbetapir imaging showed visible radiotracer uptake in the myocardium in all patients (Figure 1) with mean SUV 2.3 ± 0.4. Patient 7 was asymptomatic from a cardiac perspective with normal levels of troponin T and NTproBNP, but had a positive PYP study (Table 1) in the setting of negative testing for a monoclonal protein and was considered to have cardiac amyloidosis at the time of enrollment. This patient had the highest myocardial SUV value of 3.3. Mean SUV for patients at risk was 2.3 ± 0.4, and for controls was 2.2 ± 0.3. All patients had myocardial SUV values above previously described healthy controls (SUV 1.4-1.7), and all patients had mean myocardial counts more than 2x blood pool counts (8). Blood pool, liver, and paraspinal muscle SUV values are also shown in the Table 1.

**Table 1:** Summary of patients and imaging results.

				**F18-Florbetapir**							
**Patient**	**Age**	**Sex**	**History**	**Visual LV uptake**	**Mean SUV**				**Heart/BP ratio**	**Echo IVS**	**Additional Imaging**
					**LV**	**Blood pool**	**Paraspinal**	**Liver**			
1	77	F	Smoldering myeloma	Yes	1.9	0.7	2.0	10.8	2.7	1.4 cm	CMR abnormal
2	70	M	Smoldering myeloma	Yes	2.7	0.7	2.2	10.9	3.9	1.6 cm	CMR normal
3	75	M	ATTRv - Val30Met	Yes	2.9	0.9	2.1	17.0	3.2	1.6 cm	PYP negative
4	80	M	ATTRwt	Yes	2.2	0.8	1.7	15.0	2.8	0.9 cm	PYP negative
5	72	F	ATTRwt	Yes	2.0	0.7	1.3	12.1	2.9	1.2 cm	PYP negative
6	73	F	ATTRv - Ala81Thr	Yes	1.9	0.9	3.0	15.3	2.1	0.9 cm	PYP negative*
7	67	M	ATTRwt	Yes	3.3	0.6	1.5	10.0	5.5	1.4 cm	PYP positive
8	65	M	ATTRwt	Yes	2.4	0.6	2.1	10.9	4.0	1.3 cm	PYP negative
9	83	M	CTS negative	Yes	2.5	0.9	0.8	14.4	2.8	N/a	N/a
10	67	M	CTS negative	Yes	2.3	0.7	1.6	14.8	3.3	N/a	N/a
11	66	F	CTS negative	Yes	1.8	0.8	1.3	8.3	2.3	N/a	N/a
12	69	F	CTS negative	Yes	2.0	1.0	1.2	10.4	2.0	N/a	N/a

ATTRv, hereditary transthyretin amyloidosis; ATTRwt, wild-type transthyretin amyloidosis; BP, blood pool; CA, cardiac amyloidosis; CTS, carpal tunnel surgery biopsy; IVS, interventricular septum; LV, left ventricle; PYP, technetium pyrophosphate; SUV, standardized uptake value. *Developed Grade 3 uptake of PYP scan 3 years later.

Of the seven patients with a potential for cardiac involvement, two had smoldering myeloma (1 IgG lambda with free light chain difference 873 mg/L, 1 IgG kappa with free light chain difference 339 mg/L), and five had ATTR with negative PYP scans (four with TTR deposits in the tenosynovium (7) and one with hereditary ATTR polyneuropathy). Patient 1 was asymptomatic from a cardiac perspective but had an abnormal cardiac MRI with native T1 time 1,300 msec (but without late gadolinium enhancement), an NTproBNP above reference range (607 pg/mL), but no fat pad or bone marrow biopsy evidence of amyloid deposits.

Patients were followed clinically for 3 years, and none developed new symptomatic cardiac amyloidosis. Two patients had follow-up PYP scans (Figure 1); patient 6 had Ala81Thr mutation and developed asymptomatic PYP conversion to Grade 3 uptake 3 years after enrollment despite initial low F18-florbetapir myocardial retention. Interestingly, F18-florbetapir muscle uptake in the cohort was highest in this patient.

## Discussion

In this prospective pilot study, F18-florbetapir identified qualitative and quantitative radiotracer uptake above previously described control levels in all 12 patients. The one patient with technetium PYP evidence of cardiac amyloidosis did have the highest quantified myocardial SUV values. No patients developed clinical evidence of cardiac amyloidosis over 3 years, but one patient had asymptomatic conversion to a positive PYP scan despite low F18-florbetapir uptake at baseline.

This pilot study raises several important questions about cardiac amyloidosis, progression, and the utility of molecular PET imaging for this disease. One of the most important points to make relates to the comparison of F18-florbetapir with SPECT-based PYP and other bone scintigraphy. PYP has the benefit of being interpreted qualitatively on reconstructed tomographic images, while F18-florbetapir does not. That is to say, if SPECT PYP images demonstrate any radiotracer localized to the myocardium, i.e., highly specific for cardiac amyloidosis (and most likely ATTR). Yet, all patients undergoing F18-florbetapir PET have identifiable radiotracer localized to the myocardium that is significantly above blood pool values. Thus, specific thresholds are needed to separate positive from negative cases. Other protocols using a retention index could be examined in early disease, though these protocols involve up to a 60-min scan duration (6, 10) which may be difficult for some patients and inefficient for lab throughput. Our protocol utilized SUV calculation at a fixed time point 10–20 min after radiotracer injection which was based off of an analysis demonstrating good correlation between retention index and SUV measurements (8), and is in line with other PET protocols which generate static images for infection and inflammation. As only small differences were noted in F18-florbetapir myocardial SUV values, the utility of this protocol in the detection of early cardiac amyloidosis using this protocol appears limited.

Additionally, the speed of progression of myocardial amyloid deposits is currently unknown. AL amyloidosis has been thought to progress much more rapidly than ATTR amyloidosis; however, patient 6 had progression of ATTR cardiac amyloidosis on PYP nuclear scintigraphy over 3–4 years which was not preempted by significant F18-florbetapir uptake. As both patients during the study period with positive PYP scans were asymptomatic, this confirms the high sensitivity of PYP in early asymptomatic disease (11).

At this time, PET imaging with F18-florbetapir in cardiac amyloidosis remains an imaging modality best used in clinical research until more data accumulates regarding the best protocol to maximize sensitivity, specificity, differentiation among amyloid subtypes, and ease of interpretation. Other PET radiotracers such as the thioflavin analogs F18-florbetaben, F18-flutemetamol, and C11-Pittsburgh compound B, and bone-seeking agents targeting microcalcifications like F18-NaF are similarly under investigation (12).

This pilot study is limited by an insufficient sample size to make broad conclusions about the utility of molecular PET imaging in cardiac amyloidosis. Further study is needed in asymptomatic patients or those with early disease, in both the AL and ATTR subtypes, to expand upon these findings. Repeat imaging could shed light on subclinical progression of disease and is an area of future research. At this time, PET imagings is not ubiquitously available and is more costly than technetium-based SPECT bone scintigraphy. In addition, this study also raises additional questions regarding the potential false negative rate of tenosynovial biopsy in our “control” patients. While these patients had negative tenosynovial biopsies and no signs or symptoms of amyloidosis, though unlikely, low level amyloid infiltration could have existed in the myocardium.

## Conclusion

In this prospective pilot study, F18-florbetapir identified radiotracer uptake in the myocardium in early cardiac amyloidosis, patients with a potential for developing cardiac involvement of amyloidosis, and controls. F18-florbetapir uptake was qualitatively noted in the myocardium above blood pool in all patients, and there was significant overlap among quantitative uptake. There was no difference in the intensity of F18-florbetapir uptake in at-risk patients and controls. Future studies should investigate metabolic PET tracers and protocols for early detection of cardiac amyloidosis.

## Data Availability Statement

The raw data supporting the conclusions of this article will be made available by the authors, without undue reservation.

## Ethics Statement

The studies involving human participants were reviewed and approved by Cleveland Clinic. The patients/participants provided their written informed consent to participate in this study.

## Author Contributions

BS and WJ conceived of the idea. BS, MH, WJ, and FD created the study design and planned the research. BS, AB, and JD carried out the research experiments. BS wrote the first draft of the manuscript. All authors critically reviewed the manuscript.

## Conflict of Interest

BS is a consultant for Alnylam and Pfizer and has received research funding from Pfizer. MH has served on advisory boards for Alnylam, Eidos, AKCEA, and Pfizer. The remaining authors declare that the research was conducted in the absence of any commercial or financial relationships that could be construed as a potential conflict of interest.
